# Health Resorts and Summer Vacations

**Published:** 1919-05

**Authors:** 


### TEXAS MEDICAL JOURNAL

DR. F. E. DANIEL,
Founder.

Established July,
1885

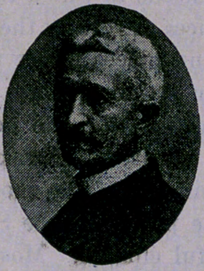

MRS. F. E. DANIEL,
Publisher and Managing
Editor.

VOL. XXXIV NO. 10
AUSTIN,
APRIL, 1919.

PUBLISHED MONTHLY.
Subscription:
$1.50

The publisher is not responsible for views of contributors.

ASSISTANT EDITORS:

DR. JOHN S. TURNER, Dallas, Texas.
DR. E. B. PARSONS, Palestine, Texas.
DR. A. P. HOWARD, Houston, Texas.
DR. JOE BECTON, Greenville, Texas.
DR. M. F. BLEDSOE, Port Arthur.
DR. J. H. FLORENCE, Tulsa, Oklahoma.
DR. J. H. REUSS, Cuero, Texas.

### EDITORIAL.

### Health Resorts and Summer Vacations.

Under the advice of their physicians the people of this country are learning to conserve their health and strength. They are finding that they cannot work all the year through without a rest and still do the best work. Almost every person you meet—men and women—are suffering more or less from some physical ailment. It may not be anything serious at first, but unless it is looked after, in time it will make severe inroads upon the general health. In nearly every case this trouble is due to over work or to worry. It comes from staying too continuously at one pursuit without rest. The work itself may not be hard, it may not be exacting, but the monotony of it from month to month and year to year gets on one's nerves after a while.

The doctors are learning, when one comes to them with symptoms of nervous troubles, to ascertain just what is back of the trouble, and having learned this they prescribe the necessary remedy, which in most cases is a change of climate or a vacation and rest, or the treatment that can best be given in some well equipped sanitarium.

As a result of this, resorts and sanitariums are springing up all over the country and those that are progressive and alert in letting their facilities be known are doing a business of a magnitude scarcely conceivable a few years ago. These vary in character according to the needs to which they cater. Some of them are little more than places of rest by the seashore or in the mountains; some are watering places where mineral waters of different kinds, all more or less medicinal, are featured; some are bathing places where restorative and health giving baths under medical direction are featured. Eureka Springs, Arkansas, in the Ozark mountains, is one of the most popular mountain resorts frequented by the people of the Southwest, because of its proximity and delightful climate. Most of the resorts are provided with hospitals and sanitariums for the care of those who need not only rest, but medical attention. Places of this kind in this state that are coming into prominence are Marlin, where the Torbett Sanitarium is located, and Kerrville, where the Secor Sanitarium is achieving a reputation in this state somewhat like the Battle Creek Sanitarium has throughout the country.

Outside of this state there is at Battle Creek, Michigan, the famous Battle Creek Sanitarium, the pioneer institution of its kind and still the leading rest place of the country where rest, recreation and recuperation are prescribed under scientific medical direction. This is the most pretentious and the best equipped place of the kind in the country and hundreds go there from Texas and adjoining states every year. A popular kind of rest place that is growing in favor every year because of real merit is the mud bath resort like Mudlavia at Kramer, Indiana, where many people obtain substantial benefit from both rest and treatment.

There are a number of places throughout the country that make a specialty of nervous and mental disorders, and where physicians send their patients who are beyond that stage where rest is considered equally important with treatment. In this state prominent sanitariums of this class are Moody's Sanitarium at San Antonio, Greenwood's Sanitarium at Houston and the Oaks Sanitarium at Austin, all of which have attained wide reputations both in and out of the state. The Punton Sanitarium at Kansas City is a large institution for nervous people that features the "home" atmosphere of the place, and is becoming well-known all over the Southwest.

Many physicians in the general practice are recommending that their patrons, even though apparently well, should have a "looking over" every once in a while in order to detect the first inroads of disease. There are places throughout the country, where, although surgical treatment is given to those who need it, special attention is given to diagnosis by a staff of expert diagnosticians, who are skilled in detecting incipient troubles. As a rule such patients, unless they require immediate surgical attention of a kind that the institution is better prepared to give, are sent back to their physicians for treatment. One of the largest and best equipped places of this kind in the South is the Temple Sanitarium with its staff of physicians and surgeons. It has been called "The Mayo Clinic of the South," because of the character of work done there and the general facilities for diagnosing and handling difficult cases. Dr. Kenney's Sanatorium at San Antonio, has long ejoyed a reputation among physicians for its splendid equipment and expert staff for handling surgical and obstetrical cases. It also maintains a department for the treatment of those addicted to the use of alcohol and drugs.

There are scores of hospitals springing up all over the Southwest where staffs of experts are prepared to give the best surgical and medical attention to patients. One of the most prominent of these is the Physicians and Surgeons Hospital at Austin, a young institution that has been filled with patients from the date of its opening. The facilities afforded by these hospitals for handling all classes of surgical and medical cases have added greatly to the improvement of the general health of the people.

When people everywhere learn that if they would live long and well they must take care of themselves, and that they can't do so without a reasonable amount of rest and professional attention to their physical needs, regular medical examinations and vacations spent under the advice of physicians will be regarded as just as essential as sleep or proper dieting.

